# Spatiotemporal dynamic characteristics of typical temperate glaciers in China

**DOI:** 10.1038/s41598-020-80418-7

**Published:** 2021-01-12

**Authors:** Wang Shijin, Che Yanjun, Wei Yanqiang

**Affiliations:** 1 Yulong Snow Mountain Glacier and Environment Observation and Research Station/State Key Laboratory of Cryospheric Sciences, Northwest Institute of Eco-Environment and Resources, Chinese Academy of Sciences, Donggang West Road 320, Lanzhou, 730000 Gansu China; 2 College of Resources and Environment, University of Chinese Academy of Sciences, Beijing, 100049 China; 3 Yichun University, Yichun, 336000 Jiangxi China

**Keywords:** Climate sciences, Environmental sciences

## Abstract

China’s temperate glaciers have a relatively warm and humid climate and hydrothermal conditions at low latitudes. Temperate glaciers, however, have larger ablation, higher ice temperatures, relatively fast movement speeds, and a significant sliding process at the bottom. As a result, these glaciers are more significantly affected by climate change. On the basis of topographic maps, aerial photography, and Landsat OLI images, and combined with existing research results, this paper systematically analyzed the temporal and spatial dynamic characteristics of typical temperate glaciers. The results are as follows: (1) From the 1950s to the 1970s, compared with other types of glaciers, temperate glaciers showed strong retreat and ablation trends in terms of area, length, speed, and mass balance. (2) The reduction rates of glacier areas of Kangri Garpo, Dagu Snow Mountain, Yulong Snow Mountain (YSM), and Meili Snow Mountain (MSM) in China’s temperate glacier areas all exceeded 38%, which was far above the national average of 18% from the 1950s to the 2010s. (3) The recent length retreat rates of Azha Glacier, Kangri Garpo, and Mingyong Glacier, MSM, Hailuogou Glacier (HG), Gongga Snow Mountain (GSM), and Baishui River Glacier No. 1 (BRGN1), YSM were above 22 m/a, which was faster than the retreat rates of other regions. (4) Consistent with glacier retreat, temperate glaciers also had a faster ice flow speed. The ice flow velocities of the BGN1, HG, Parlung River Glaciers No. 4 and 94, and Nyainqêntanglha were, respectively, 6.33–30.78 m/a, 41–205 m/a, 15.1–86.3 m/a, and 7.5–18.4 m/a, which was much faster than the velocity of other types of glaciers. (5) Mass loss of temperate glaciers was most dramatic during the observation period (1959–2015). The annual mass balance from eight typical temperate glaciers fluctuated between − 2.48 and 0.44 m w.e., and the annual average change rate of mass balance (− 0.037 m w.e./a) was much higher than that in China (− 0.015 m w.e./a, *p* < 0.0001) and globally (− 0.013 m w.e./a, *p* < 0.0001).

## Introduction

**Figure 1:**
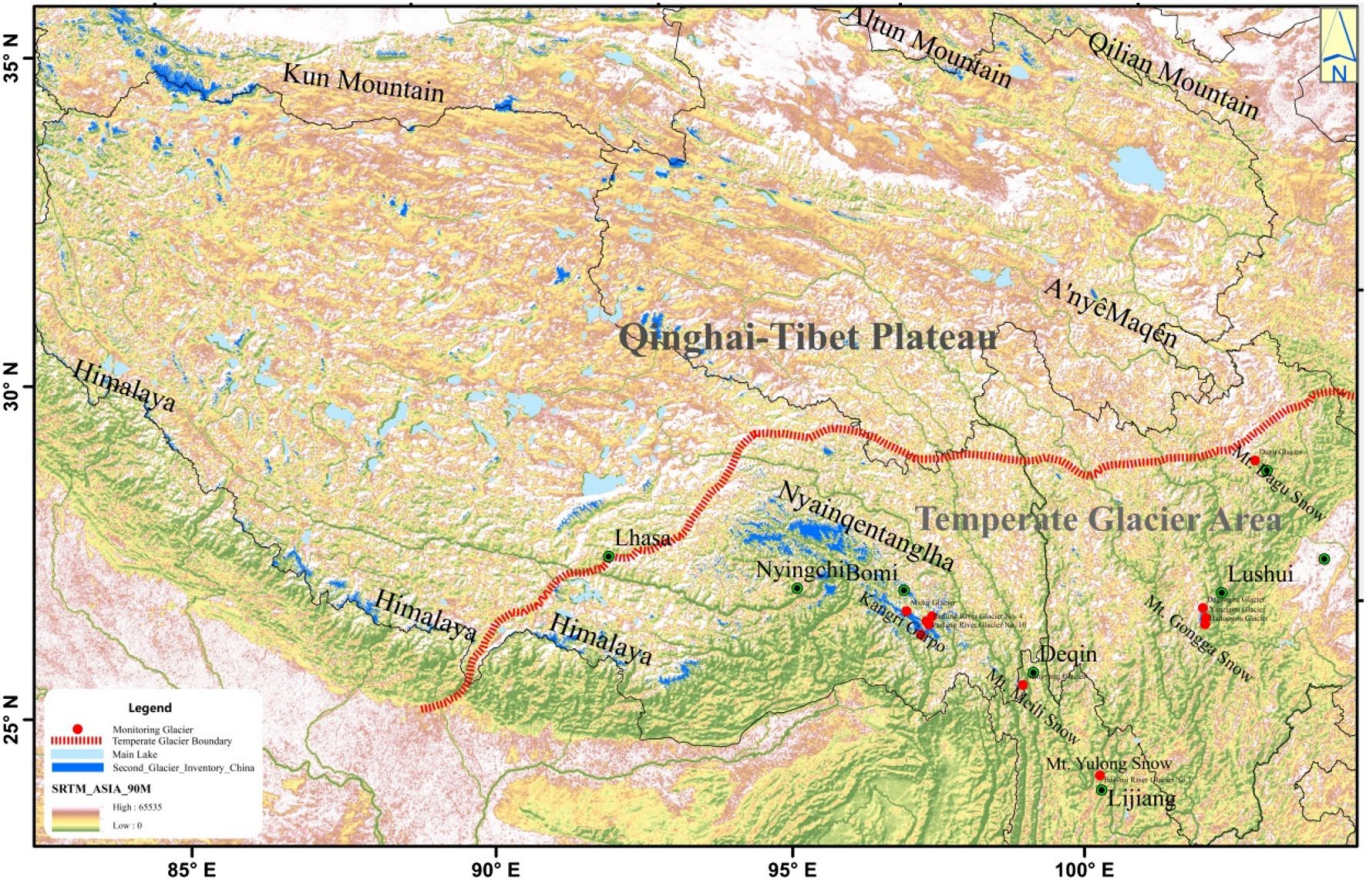
The range of China’s temperate glaciers and the prevailing airflow affecting the region. The final map was created by ArcMap version 10.2 (http://desktop.arcgis.com/en/arcmap/).

According to glacier development conditions and physical properties, China’s glaciers can be divided into three types: temperate glacier (also known as monsoon temperate glacier), subcontinental glacier, and polar continental glacier^1,2^. Among these three types, the average temperature near the mass balance line of temperate glaciers generally is above 1 °C in the summer, and the ice temperature of active layer is usually above − 1 °C, which is close to the melting point^3^. Temperate glaciers are very sensitive to variations in temperature and precipitation, and thus represent a good indicator of climate change^4^. China’s temperate glaciers are concentrated in southeastern Tibet Autonomous Region (TAR), northwestern Yunnan Province, and western Sichuan Province. This area is located in the “triangle area” between the Western Pacific Ocean, the Indian Ocean, and the Qinghai-Tibet Plateau (QTP), and it is one of the typical areas of sea-land-air interaction. The atmospheric circulation pattern affecting this area is relatively complex. The region is affected by the southwest and southeast monsoons in the summer and the southern branch of the upper-westerly circulation in the winter (Fig. 1). Under the influence of southeast and southwest monsoons, the precipitation in this area is abundant, especially in the high-altitude area, and the annual rainfall can reach 1000–3000 mm. The abundant rainfall and vapor sources are important conditions for the development of a large number of temperate glaciers in the southern Himalayas, the middle-eastern part of the Nyainqêntanglha, Kangri Garpo (Gangrigabu), Boshula Mountain, Meili Snow Mountain (MSM), Gongga Snow Mountain (GSM), Yulong Snow Mountain (YSM), and Dagu Snow Mountain (DSM)^5–7^. Compared with the other two types of glaciers, the end of the temperate glaciers often extends to the forest areas, where the vertical distribution of plants is extremely distinct, and the accessibility of glaciers is better than the accessibility of the others. In addition, these areas are blessed with a pleasant climate, a diverse environment of multiethnic and multicultural integration, and rich tourism resources. Such areas are the most attractive destinations because they feature integrated cryosphere landscapes and human activities. These temperate glaciers, however, are showing signs of accelerated melting because of their strong accumulation and melting, high temperature, fast movement, and violent bottom sliding^8^. In addition, the remarkable vertical height differences and strong quaternary glaciation in such areas are related closely to the evolution of regional human activities, the ecological environment, and the development of disasters. Many glacial lakes are located in front of the glaciers in these areas. Because of the deep rock basins in the glacial trough and valley and the high content of silt, sand, and clay in the moraine dams and their bank slopes, glacial floods and debris flows, glacial lake outburst disasters, and secondary disasters often occur.

The comprehensive study of the interaction of temperate glaciers with climate and environment can deepen our understanding of the dynamic characteristics of these temperate glaciers and their impact on sustainable economic and social development^7,9–12^. The comprehensive study of temperate glaciers is significant not only for the scientific theory of climate change but also for the social application value related to sustainable regional development. Previous studies on temperate glaciers in China have been scattered and the research started in the 1950s^13^, however, focusing either on a single glacier, on a single element, or on one or more typical glacier areas, and rarely have systematically and comprehensively analyzed the entire temperate glacier area as a whole. This early research and results accumulated valuable comparative data for the later study of temperate glacier changes^8,14–18^. Therefore, on the basis of topographic maps, aerial photography and Landsat images, and combined with previous research results, the study revealed the temporal and spatial characteristics of China’s temperate glacier change (e.g. the length, area, speed and mass balance change of glaciers) and put forward some new ideas and understandings of accelerated temperate glacier changes.

## Study area, data and methods

### Research area

China’s temperate glaciers are distributed mainly in the Hengduan range in the southeastern QTP, the southern part of the Himalayas, and the middle east of the Nyainqêntanglha, mainly in the three provinces of Tibet, Yunnan, and Sichuan. According to Chinese glacier inventory, China has 8607 temperate glaciers, covering an area of 13,203.2 km^2^, accounting for 18.6% and 22.2% of the total number and total area of glaciers in China, respectively^19^. China’s temperate glaciers are affected by the southwest and southeast monsoon (Fig. 1). The most notable features are as follows: abundant precipitation in the glacial region, especially in high-altitude areas, annual precipitation reaching 1000–3000 mm, and the height of snow line is low, ranging from 4200 to 5200 m, which is 800–1200 m^1,20,21^ lower than that of the continental glacier in China. The annual average temperature at the equilibrium line is higher than − 6 °C, the summer temperature is between 1 and 5 °C, and the glacier ice temperature is between 0 and − 4 °C, which is often lower than − 1 °C, making the glacier ablation intensity relatively large. In addition, because of the rich supply and high ice temperatures, these temperate glaciers are moving at a relatively fast speed, reaching more than 20 meters per year, and often are accompanied by bottom sliding. Because of this, the slight fluctuations in temperature can cause the temperate glaciers to move forward or retreat.

China’s temperate glaciers are concentrated primarily in Kangri Garpo, MSM, YSM, GSM, Queer Mountain, DSM, Xuebaoding, and the middle east of Nyainqêntanglha. Among them, the Kangri Garpo (29° 10′–29° 90′ N; 95° 50′–97° 95′ E) is located in the northeast of the Yalung River in Linzhi City, TAR, with a balance line height of 4900–5400 m. The main peak, Ronnie Peak (Bairiga), is 6882 m, northwest-southeast, with a total length of 280 km and the area of about 9000 km^2^. MSM (28° 11′–28° 40′ N; 98° 36′–98° 52′ E) is located in the Nushan Mountains on the southeastern edge of the QTP, between the Lancang River and the Nujiang River. It is 40 km long from north to south and covers an area of about 346 km^2^. The highest peak in this area, Kawagebo Peak, is 6740 m above sea level, and the lowest is 2020 m above sea level on the Lancang River surface, with a relative height difference of 4720 m. It is one of the regions with the richest biodiversity in the temperate zone of China and the world. The altitude of the main fan steep peak of the YSM (27° 10′–27° 40′ N; 100° 9′–100° 20′ E) is 5996 m, which is located in the southeastern edge of the QTP and covers an area of about 960 km^2^. It is the closest glacial area to the equator in the Eurasian continent (except Chaya Peak). The highest altitude of GSM (29° 20′–30° 20′ N; 101° 30′–102° 15′ E) is 7514 m above sea level. It is located in the middle section of Great Snow Mountain in the transitional zone from the Sichuan Basin to the QTP, with an area of about 10,000 km^2^. The Hailuogou Glacier (HG) on the eastern slope of the GSM is the largest glacier in its snow-capped mountains. DSM (32° 8′–32° 17′ N; 102° 1′–102° 49′ E) is located in the transitional area from the QTP to the Sichuan Basin. The altitude is between 3800 and 5273 m, the altitude of the main peak is 5273 m, and the area is about 120 km^2^.

Generally speaking, China’s temperate glaciers are controlled by a subtropical mountain and monsoon climate. Additionally, the “channel” effect of the vertical ridge valley (for example Yarlung Zangbo, Jinsha, Nu and Lancang rivers) of the southeastern QTP that enters China is the source of abundant mass supply for temperate glaciers. The plateau monsoon generated by the thermodynamic action of the QTP strengthens the intensity of the monsoon circulation from the southwest region of the Hengduan range in summer^21^. Under these conditions, China’s temperate glacier areas, such as Kangri Garpo, the middle east of Nyainqêntanglha, MSM, and YSM, have become important channels for the Indian Ocean monsoon to transport water vapor to the QTP and the wettest areas of the QTP.

### Data and methods

#### Ground observation data and processing

Mass balance is the most important monitoring elements in traditional glacial observation. At present, several temperate glaciers have been continuously monitored for glacier mass balance in the southeastern region of the QTP, including the HG of the GSM, the Baishui River Glacier No. 1 (BRGN1) of the YSM, and Parlung River Glacier No. 94 (PRGN 94). The mass balance is calculated using the records of stakes and snow pits, and then results are projected to large-scale topographical maps, which are called the contour method^22,23^. The point mass balances are calculated to glacier-wide mass balance (*B*_*a*_) using the glacier means area *S* over the same time-span, as follows:

1 $${B}_{a}=\frac{\sum\nolimits_{i=1}^{n}{b}_{i}{S}_{i}}{S}$$

where *n* is the number of interval isolines of mass balance, and *b*_*i*_ and *s*_*i*_ are the mean mass balance and area between adjacent isolines, respectively. Yang et al.^24^ reported that the annual mass balance derived from the two different contour methods were very close in Urumqi River Glacier No. 1 of east Tien Shan. In this study, the calculation method of the annual mass balance of the BRGN1) is shown in the reference by^8^, and annual mass balance data of other typical glaciers comes from the references^11,12,16,25,26^.

It is easier to monitor changes in glacier fronts than the mass balance. In general, at the end of the accumulation season and the end of the ablation season, the glacier fronts are measured and recorded by the Global Positioning System (GPS) with high accuracy. On the basis of continuous records of the glacier terminus, the fronts of these glaciers can be observed during the study period. In this paper, observation data for the GSM glacier were provided by the Gongga Mountain Alpine Ecosystem Observation and the Experiment Station of the Chinese Academy of Sciences. Ground observation data for the YSM glacier were provided by the Yulong Snow Mountain Glacier and Environmental Observation Research Station of the Chinese Academy of Sciences.

#### Remote sensing and processing

To assess the glacier outlines in the study area for different periods, we used Landsat level 1 terrain-corrected images, which we downloaded from the U.S. Geological Survey (USGS; https://earthexplorer.usgs.gov/). The images were from Landsat5 TM, Landsat 7 ETM+, and Landsat 8 OLI for the period from the 1970s to 2017 (The source of remote sensing image is attached in the supplementary table). The ETM+ images had gaps of missing data because of the instrument failure of Landsat 7’sensor. We used the landsat_gapfill.sav tool to fill in the data gaps for the ETM+ images based on the ENVI software^27^. In order to avoid the interference of cloud and snow on image interpretation accuracy, all satellite images required less than 10% cloud cover and were limited to the ablation season between June and October in the 1970s and 2010s. In order to exclude the influence of surface moraines on glacier boundary identification, we used the Google Image in 2015 provided by Google Earth as the reference for the glacier outlines of LandSat 8 OLI in 2013 and 2017. At the same time, we also modified the outline by manual visual interpretation based on field observation experience.

As for the multiband Landsat images, the method of spectral-band ratio has been demonstrated as a simple, highly efficient, and accurate technique to extract outlines of glaciers^28,29^. These images can decrease such problems as sensor saturation, shadowed areas, and discriminate debris mantled ice and ice marginal water bodies^30^. In this work, we used Landsat spectral bands to calculate the band ratio (Eq. 1): Ratio = CH_n_/CH_m_ (1) where n is the band number of the *Red spectral* (Red) band (Band 3 in TM/ETM+, Band 4 in OLI) or the *Near-infrared spectral* (NIR) band (Band 4 in TM/ETM+, Band 5 in OLI), and m is the band number of *Short-wave infrared spectral* (SWIR 1) band (Band 5 in TM/ETM+, Band 6 in OLI). To eliminate snow-cover influences, the normalized difference snow index (NDSI) is used to distinguish the snow zones (Eq. 2):

2 $$NDSI=({\mathrm{CH}}_{n}-{\mathrm{CH}}_{m})/({\mathrm{CH}}_{n}+{\mathrm{CH}}_{m})$$

where *n* is the band number of the visible spectral band, and *m* is the band number of the near-infrared spectral band, e.g., Band 2 and Band 5 in TM/ETM+, Band 3 and Band 6 in OLI. This is based on the difference between the strong reflection of visible radiation and the near-total absorption of shortwave infrared wavelengths by snow. The NDSI has been effective in distinguishing snow from similarly bright soil, vegetation and rock, as well as from clouds in Landsat imager^6^.

In terms of accuracy verification, we estimate this error term based on a buffer for each glacier similar to the method suggested by Pfeffer et al.^31^ and Tielidze^32^ with a buffer size 15 m (half of the image pixel size) for all aerial images and maps, based on the 30 m image pixel size, and map uncertainty in the absence of stated historical accuracies. The error is estimated by:

3 $$\mathrm{Uncertainty}=\left({{\Delta }_{buffer}}/{{\Delta }_{glacier}}\right)*100\%$$

where uncertainty (%) is the area error determined for each glacier, Δ_*buffer*_ is the area of an outline buffer around each glacier, Δ_*glacier*_ is the area of the glacier.

#### Climate reanalysis data

The reanalysis data was obtained from the National Centers for Environmental Prediction/National Center for Atmospheric Research (NCEP/NCAR) Reanalysis 1 from 1958 to 2016 (can be downloaded at https://www.esrl.noaa.gov/psd/data/gridded/data.ncep.reanalysis2.html).

## Temporal and spatial dynamic characteristics

### Glacier area change of typical mountain areas

**Figure 2:**
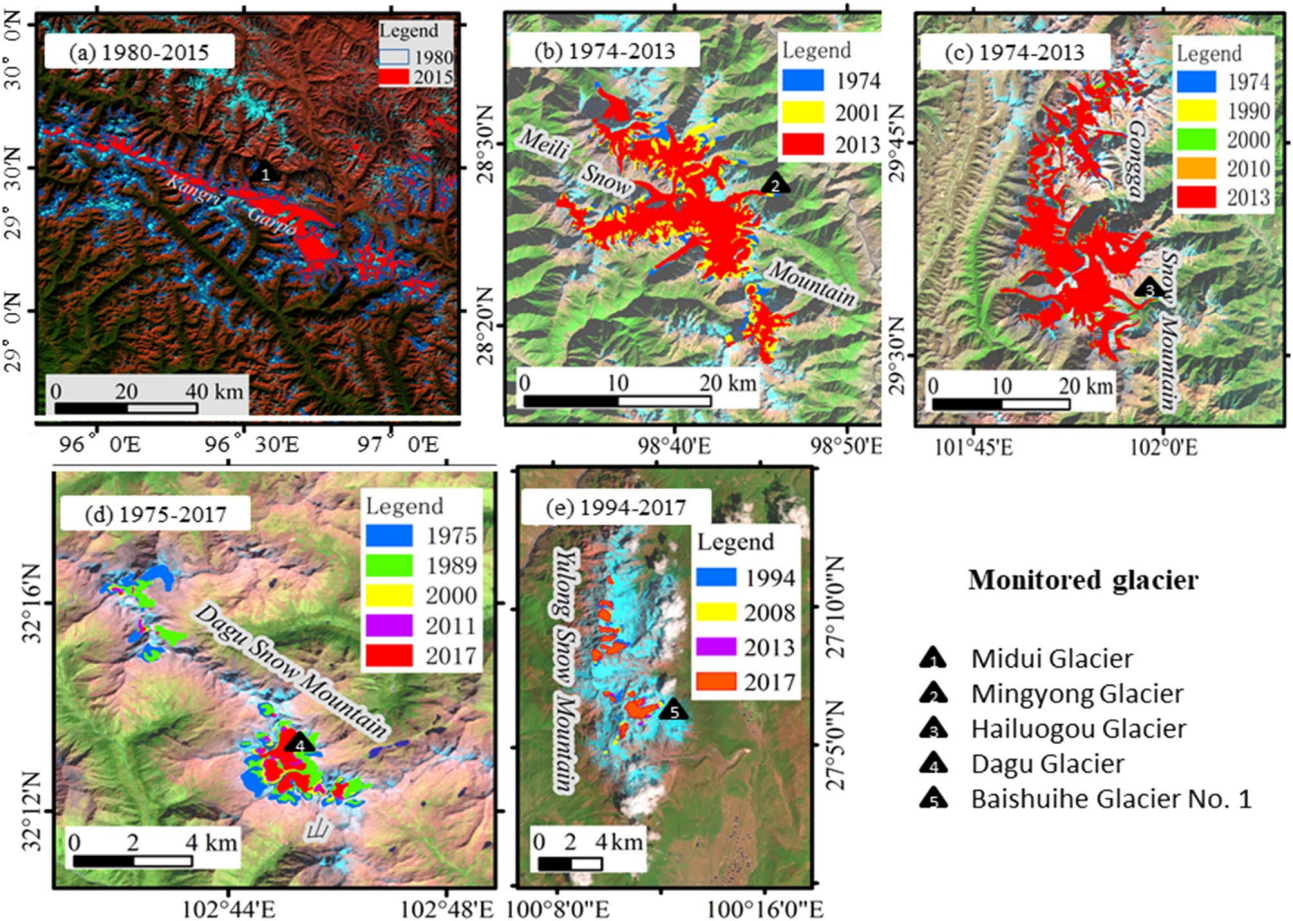
Spatial distribution of glaciers change on Kangri Garpo (**a**), Meili (MSM) (**b**), Gongga (GSM) (**c**), Dagu (DSM) (**d**) and Yulong Snow Mountains (YSM) (**e**) during different periods. The satellite images were provided by U.S. Geological Survey (USGS; https://earthexplorer.usgs.gov). The final maps of glacier change were created by ArcMap version 10.2 (http://desktop.arcgis.com/en/arcmap/).

Kangri Garpo had 1166 glaciers, covering an area of 2048.50 km^2^ and an average glacier size of 1.76 km^2^ in 2015. During the period from 1980 to 2015, the number of glaciers decreased by 154 and the area of glaciers decreased by 679.50 km^2^ (− 24.91%). The annual average area retreat rate was 0.69%, and the average altitude at the end of the glacier increased by 111 m. Ten glaciers in the area were advancing or surging, however^33^ (Fig. 2a,Table 1). MSM distributed 47 glaciers with a area of 171.56 km^2^ in 1974. In 2001, the number of glaciers increased to 48, while the total area decreased to 156.72 km^2^. In 2013, the number of glaciers increased to 66, while the total area decreased to 123.58 km^2^. From 1974 to 2013, the area of glaciers in the MSM reduced by 47.98 km^2^, and the annual average decrease rate was − 0.97%. In addition, after 2001, the glaciers in the MSM showed a significant decrease in the number and the area of glaciers (Fig. 2b, Table 1). GSM had 74 glaciers, with a total area of 252.4 km^2^ in 1974. In 1990, the number of glaciers increased to 76, and the total area decreased to 238.47 km^2^. In 2013, the number of glaciers was 77, while the total area decreased to 220.22 km^2^. From 1974 to 2013, the area of glaciers retreated by 32.18 km^2^, and the average area decerase rate was − 0.32%/a. Especially, after 2000, the glacier area retreat trend increased significantly (Fig. 2c, Table 1). DSM had 13 glaciers in 1975 with an area of 6.84 km^2^. In 2000, the glacier area decreased to 2.09 km^2^. In 2011, the glacier area was reduced to 1.95 km^2^. In 2017, four glaciers have disappeared and total glacier area was reduced to 1.75 km^2^. From 1975 to 2017, the average glacier area decreased by − 1.73%/a on the DSM^6^ (Fig. 2d, Table 1). YSM distributed 22 glaciers with a total area of 12.45 km^2^ in 1957. In 2013, the number of glaciers decreased to 16, while the total area decreased to 4.76 km^2^. In 2017, the number of glaciers was reduced to 13, while the total area was reduced to 4.48 km^2^. From 1957 to 2017, the area of glaciers decreased by 7.97 km^2^, with an average decrease rate of 0.13 km^2^/a and average decrease rate reached 1.05%/a^8^ (Fig. 2e, Table 1).

**Table 1:** Comparison of the change of typical glacier areas in China.

Glacier area	Data (period)	Period (year)	Area change (%)	Annual change (%/a)	References
Altai Mountains	2	1960–2009	− 36. 91	− 0.74	^11,12^
Tianshan	4	1959–2010	− 18.41	− 0.35	^34^
Altun Mountain	4	1973–2015	− 16.74	− 0.39	^35^
East Pamir-West Kunlun Area	4	1976–2016	− 12.19	− 0.30	^36^
West Kunlun	2	2010–2017	33.04	4.13	^37^
Karakorum	4	1978–2015	− 7.77	− 0.20	^38^
Animaqing Mountain	3	1992–2010	− 9.04	− 0.48	^39^
Himalayas	4	1990–2015	− 10.99	− 0.42	^40^
Western Nyainqentanglha	4	1976–2011	− 20.82	− 0.58	^41^
Kangri Garpo	2	1980–2015	− 24.91	− 0.69	^33^
Meili Snow Mountain	3	1974–2013	− 38.82	− 0.97	This study
Gongga Snow Mountain	5	1974–2013	− 12.75	− 0.32	This study
Dagu Snow Mountain	5	1975–2017	− 74.42	− 1.73	^6^
Yulong Snow Mountain	5	1957–2017	− 64.02	− 1.05	^8^

Because of different periods, different sources of images and different interpretation methods, these data may differ from other documents, but the differences do not affect the rapid change of temperate glaciers.

Generally, the reduction rates of the temperate glacier areas in the Kangri Garpo, DSM, YSM, and MSM, exceeded 38% during the 1970s and 2010s, which was far above the national average of 18% in the same period^42^. The response characteristics and response time of different scales and types of glaciers to climate change were different, as was the climate change in different regions, leading to large regional differences in glacier changes in western China (Table 1).

### Length change of typical glaciers

**Figure 3:**
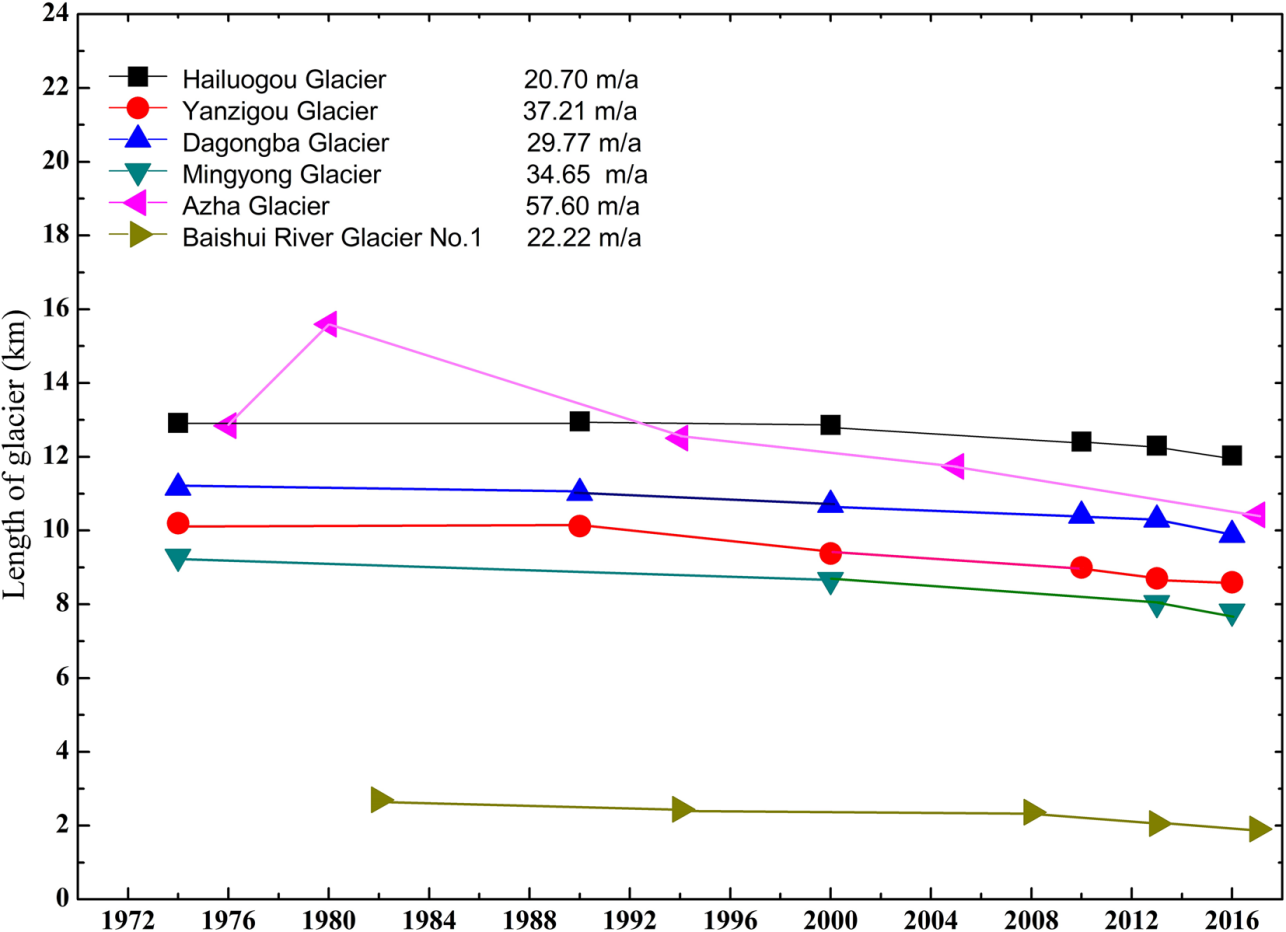
Length change of typical temperate glaciers from the 1960s to the 2010s.

Based on Landsat remote-sensing images and existing research data, combined with recent Google Earth images, we organized the dynamics of the length of six typical temperate glaciers. Since the 1970s, all six glaciers investigated exhibited notable retreat trend. During this period, Azha, Hailuogou, Dagongba, Yanzigou, Mingyong and Baishui River Glacier No. 1 (BRGN1) retreated by 2420 m, 890 m, 1280 m, 1600 m, 1490 m, and 640 m with an annual retreat of 57.60 m, 20.70 m, 29.77 m, 37.21 m, 34.65 m and 22.22 m, respectively. Moreover, the retreat rate from Azha, Yanzigou and Mingyong Glaciers were accelerating (Fig. 3, Table 2). Azha Glacier, in the Kangri Garpo area, shrunk from 12.84 km in 1976 to 10.42 km in 2017, with a retreat rate of 18.85%/a. The history of the Ata Glacier thus illustrates the largest amplitude of glacial retreat and reveals an accelerating trend in retreat since the 1990s^11,12^. The length of the Mingyong Glacier, in the MSM, retreated from 9.29 km in 1976 to 7.80 km in 2016, with a retreat rate of 16.04% and an annual retreat of about 36 m/a. According to the position of the end of the glacier in 1930, since the 1930s, the HG, GSM had shrunk by about 2 km. The glacier retreated to 12.92 km in 1974 and decreased to 12.03 km in 2016. Between 1966 and 2010, the average annual retreat rate of the HG was about 25–30 m/a. Between 1974 and 2016, the annual retreat rate was about 20 m/a. Compared with 1966–2010, the retreat rate slowed slightly, but it did not reduce the melting rate of the entire glacier surface. Compared with pictures of glaciers in 1930, it is obvious that the ablation zone of the HG was obviously thinner. Among the Landsat remote-sensing images and existing research data, Glacier No. 2 in Hailuogou had been separated from the HG^16^. The length of the BRGN1, YSM shrunk from 2.54 km in 1982 to 1.90 km in 2017, with a retreat rate of 25.20% and an annual retreat of about 24.62 m/a (Fig. 3).

**Table 2:** Frontal retreat of six typical temperate glaciers for different period.

Glacier name	Period	Length (km)	Average annual shrinkage length (m)
Azha	1976–2017	12.84 km, 1976 15.59 km, 1980 12.50 km, 1994 11.74 km, 2005 10.42 km, 2017	57.60
Hailuogou	1974–2016	12.92 km,1974 12.96 km, 1990 12.86 km, 2000 12.41 km, 2010 12.03 km, 2016	20.70
Dagongba	1974–2016	11.16 km, 1974 11.02 km, 1990 10.70 km, 2000 10.40 km, 2010 9.88 km, 2016	29.77
Yanzigou	1974–2016	10.20 km, 1974 10.12 km, 1990 9.38 km, 2000 9.00 km, 2010 8.70 km, 2013 8.60 km, 2016	37.21
Mingyong	1974–2016	9.29 km, 1974 8.66 km, 2000 7.80 km, 2016	34.65
Baishui River Glacier No.1	1982–2017	2.70 km,1982 2.44 km, 1994 2.36 km, 2008 1.90 km, 2017	22.22

Compared with other types of glaciers, the retreat trend of China’s temperate glaciers is more significant. From 1959 to 2009, the average annual retreat rate of glaciers in the Altai Mountains was 16.4 m/a. From 1942 to 2014, the average annual retreat rate of glaciers in the Tianshan Mountains was 1.5 m/a. Glaciers in the Pamir region were relatively stable during 1976 and 2016. From 1963 to 2001, the average annual retreat rate was 0.7 m/a. From 1956 to 2008, the average annual retreat rate of glaciers in the Qilian Mountains was 5.7 m/a. From 1966 to 2010, the average annual retreat rate of glaciers in the Kunlun Mountains was 4 m/a. From 1974 to 2012, the average annual retreat rate of glaciers in the Tanggula Mountains was 2.8 m/a. From 1966 to 2007, the average annual retreat rate of glaciers in the Himalayan was 6.1 m/a. From 1970 to 2007, the average annual retreat rate of glaciers in the Nyainqêntanglha was 10.6 m/a. In contrast, the Karakorum Glacier, which advanced 379 m from 1968 to 2000, had an average annual advancing speed of 11.8 m/a (the length of these glaciers was the average of all glacier lengths in the areas studied)^25^. Overall, the annual retreat rates of China’s temperate glaciers are significantly higher than those of continental and polar continental glaciers.

### Ice flow change of typical glaciers

Ice mass redistribution, water and thermal environment and dynamic balance of the glacier caused by glacier movement are the primary indicators and main mechanisms of glacier existence and development relative to other natural ice bodies. Seasonal variations in glacier velocity have often been linked to surface melt-induced basal lubrication^43–45^. Studies have shown that temperate glaciers move faster than continental glaciers of the same size^46^. Glaciers with large ice flows and speeds should have a large mass balance gradient, and glaciers with large ice flows and speeds should flow into a narrow valley bed from a wide grain snow basin^47^.

**Figure 4:**
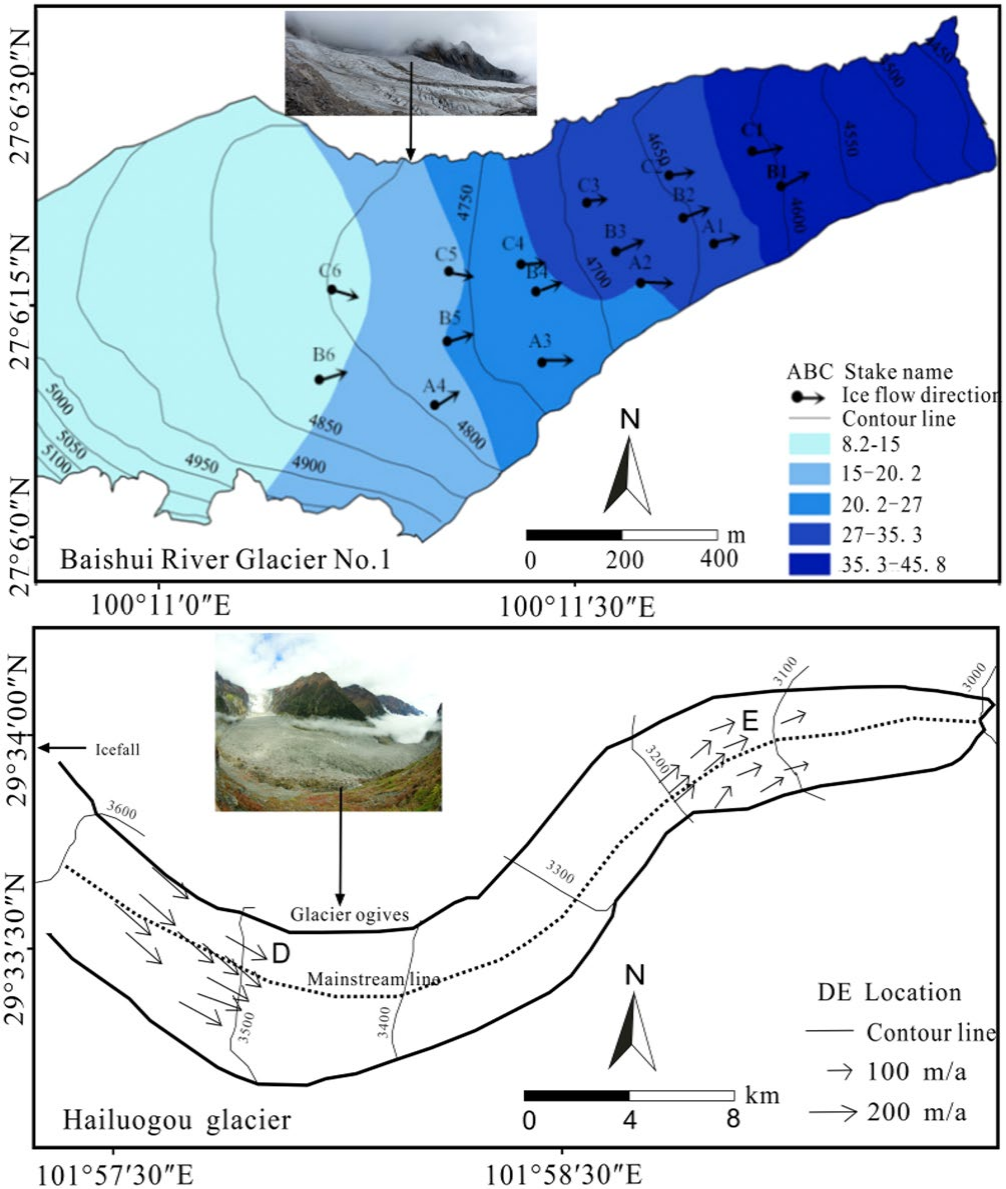
Spatial distribution for the surface flow velocity of the BRGN1 and HG.

From 2016 to 2017, the field monitoring results of the movement speed of the BRGN1, YSM showed that the maximum movement speed of the glacier was 30.78 m/a; the minimum speed was 6.33 m/a, and the annual movement speed of the whole glacier was about 16.29 m/a (Fig. 4). Especially, we used Unmanned Aerial Vehicles (UAVs) to monitor the dynamic features of velocity of the debris‐covered region of the BRG1 during 20 May and 22 September 2018. The result showed that mean displacement of debris‐covered glacier surface was 18.30 m ± 6.27 m, that is, the mean daily velocity was 0.14 m/d ± 0.05 m/d during the summer^48^. The sliding of the bottom of the glacier, the terrain under the ice, and the width of the ice surface were the main factors affecting the spatial distribution of the movement speed of the BRGN1. Glacier movement was reduced from the vicinity of the main line to the sides on the cross section, this movement gradually reduced from the end to the glacier grain snow basin on the longitudinal section, which was different from the general mountain glaciers. In the direction of glacier movement, the velocity vector mostly moved down the mainstream line or slightly deviated from the mainstream line (Fig. 4). From July to October, all of the glaciers had higher ice temperatures and more precipitation events. The surface of the glaciers melted significantly, and ice fissures fully developed. The precipitation and melt-water entered the glaciers or the bottom through the ice fissures, the refreezing rate was insignificant, and the bottom of the glaciers accelerated to slide. From October to February of the following year, the melt-water on the ice surface was reduced or even disappeared, the monsoon subsided, and precipitation was scarce and the accumulation of the glacier mass was lacking, which in turn contributed to a decrease in the movement speed of glaciers. From the end of February to May of the following year, the accumulation of glacier mass was much larger than the amount of ablation, the number of snowfalls was high, and the amount of snowfall and intensity was large. The accumulation of the glacier mass increased and glacier movement speed also increased under gravity^49,50^.

The maximum movement speed of the central part of the glacier tongue of the HG, GSM was 188.8 m/a, and the average movement speed was as high as 155.3 m/a. The upper part of the glacier tongue of the HG and the Gongba Glacier had the largest velocity, which decreased from top to bottom. The Yanzigou had the largest velocity at the end of the glacier. The tower-like distribution of the upper part of the glacier tongue and the sliding speed of the glacier were the primary reasons for this phenomenon. In the summer of 2008, the movement speed of the glacier measured by 28 sight rods in the HG ablation zone showed that the speed of glacier movement in the ablation zone increased significantly with the distance from the end of the glacier. The maximum speed of the glacier movement was 205.0 m/a, at an altitude of 3550 m in point D, and the minimum speed was 41.0 m/a, near point D of the glacier^51^. Mountain glaciers flowing through valley turns may have hindered glacier movement to some extent^52^ (Fig. 4). As early as the 1980s and 1990s, some scholars used optical theodolites to measure the speed of glacier movement in the ablation zone of the HG. The results showed that the speed of the glacier movement occurred as a result of obvious seasonal changes. Because of the rapid flow of surface melt-water to the bedrock interface to enhance sliding, the average speed in the summer was higher than that of winter^20,53^. According to the remains of the victims of the Sino-Japanese mountaineering team, ice flow speed of the Mingyong Glacier, MSM reached 533 m/a^54^. In 2006–2007, the maximum annual ice flow velocity of the Parlung River Glacier No. 4 (PRGN 4) was 86.3 m/a, and the velocity gradually reduced to 15.1 m/a toward the end of the ice tongue. The annual maximum surface ice flow velocity of the PRGN 94 was 18.4 m/a, which also occurred in the upper-middle part of the glacier tongue, and gradually decreased to 7.5 m/a at the end of the glacier tongue. The direction of the movement velocity was consistent with the direction of the main line of the glacier, which was characterized by the movement characteristics of the general valley glacier. The two glaciers were located in the same area, and the reason for the difference in the movement speed was that the glaciers were different sizes. PRGN 4 was larger than PRGN 94^46^.

### Mass balance change of typical glaciers

**Figure 5:**
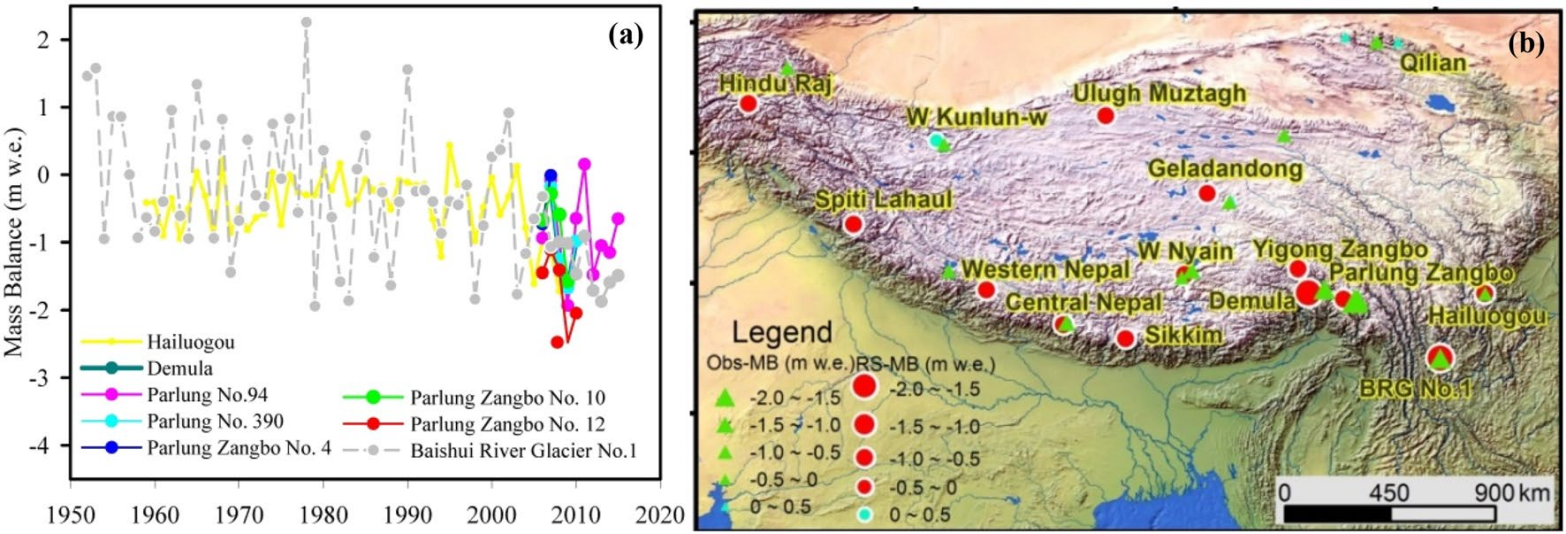
Time (**a**) and space (**b**) difference of recorded mass balance of typical temperate glaciers. (The triangle and circle in the bottom figure indicate glacier mass balance based on ground observation and remote-sensing monitoring, respectively.). (**a**) Created by Origin Pro 8 SR0 v8.0724 (b724) (www.OriginLab.com); (**b**) Created by ArcMap version 10.2 (http://desktop.arcgis.com/en/arcmap/).

We recorded the mass balance of eight glaciers (i.e. HG, BRGN1, PRGN 4, 10, 12, 94, 390, and Demula Glacier) for more than four year in China’s temperate glacier region. The observation records showed that the annual mass balance of the HG varied from − 1.716 to 0.436 m w.e. in 1959–2008, and the annual average mass balance was − 0.442 m w.e. (the measured mass balance ranged from − 1.215 to 0.436 m, and the average mass balance was − 0.336 m w.e. in 1988–1999). The mass balance of Demula Glacier fluctuated between − 1.673 and 0.170 m w.e. in 2006–2010, and the annual average mass balance was − 1.018 m w.e. The PRGN 4, 10, 12, 94, 390 of the Nyainqêntanglha were centrally monitored during the period from 2005 to 2010. Their annual mass balance varied between − 2.476 and 0.155 m w.e, and the mass loss was severe. The annual mass balance of the BRGN1, YSM, ranged from − 0.907 to − 1.872 mm w.e. during the period from 2008 to 2017. The variation in the annual mass balance of the BRGN1 was significantly higher than that of the HG, GSM and the Parlung River Glaciers (Fig. 5a)^8,55^.

The topography and climatic characteristics of the regions covered by temperate glacier are different, which lead to differences in glacial ablation. On the basis of the typical glacier mass balance observations of the QTP, monitoring the glacial mass balance of 13 glaciers over one year, and using the results of research on glacier mass balance in typical mountain areas monitored by remote-sensing data, we calculated the annual average mass balance of each glacier or mountain glacier (Table 3). These mass balance monitoring records showed that the loss of the temperate glaciers (Demla, PRGN 10, 12, 94, Zhuxi Glacier) in the southeastern part of the QTP was more serious than the loss of other continental glaciers (Namunani, Muztag Peak, Qiyi and Small Dongkemadi Glacier) (Table 3). In addition, almost important, the ablation or mass loss of the Demla, PRGN 10, 12, 94, Zhuxi Glaciers was much stronger than that from the surrounding regions (Fig. 5b, Table 3).

**Table 3:** Glacier mass balance information based on ground observation^58^ and remote sensing monitoring on the QTP^55^.

Glacier name	Mass balance	Ground monitoring			Remote-sensing monitoring		
		Lon	Lat	Period	Name of glacier area	Mass balance	Period
Palong Glacier No. 4	− 0.37 ± 0.36	96.98	29.39	1980–2015	Hindu Raj	− 0.11 ± 0.13	1973–2000
Hailuogou Glacier	− 0.34 ± 0.44	101.92	29.58	1989–1999	Spiti Lahaul	− 0.04 ± 0.1	1973–2000
Demla Glacier	− 1.02 ± 0.55	97.02	29.36	2007–2010	West Kunlun	0.02 ± 0.1	1973–2000
Palon Glacier No. 94	− 0.90 ± 0.56	96.98	29.39	2006–2016	Mudu Tag	− 0.06 ± 0.12	1975–2000
Kangwure Glacier	− 0.51 ± 0.23	85.82	28.47	1992–2010	West Nepal	− 0.23 ± 0.18	1975–2000
Namunani Glacier	− 0.42 ± 0.16	81.28	30.45	2006–2010	Central Nepal	− 0.28 ± 0.11	1974–2000
Muztag Peak Glacier	− 0.18 ± 0.41	75.06	38.24	2006–2010	Sikkim	− 0.30 ± 0.12	1975–2000
Guren River Glacier	− 0.31 ± 0.47	90.24	30.21	2006–2010	Nyainqentanglha	− 0.25 ± 0.15	1975–2000
Palong Glacier No. 10	− 0.78 ± 0.48	96.90	29.29	2006–2009	Geladandong	− 0.22 ± 0.12	1976–2000
Palong Glacier No. 12	− 1.70 ± 0.49	96.90	29.30	2006–2010	Yigong River	− 0.11 ± 0.14	1975–2000
Zhuxi Glacier	− 0.52 ± 0.57	91.45	30.87	2008–2010	Palung River	− 0.19 ± 0.14	1974–2000
Qiyi Glacier	− 0.18 ± 0.37	97.76	39.24	1975–2010			
Small Dongkemadi Glacier	− 0.27 ± 0.38	92.06	33.08	1989–2010			

## Discussion

### Some rapid change features

**Figure 6:**
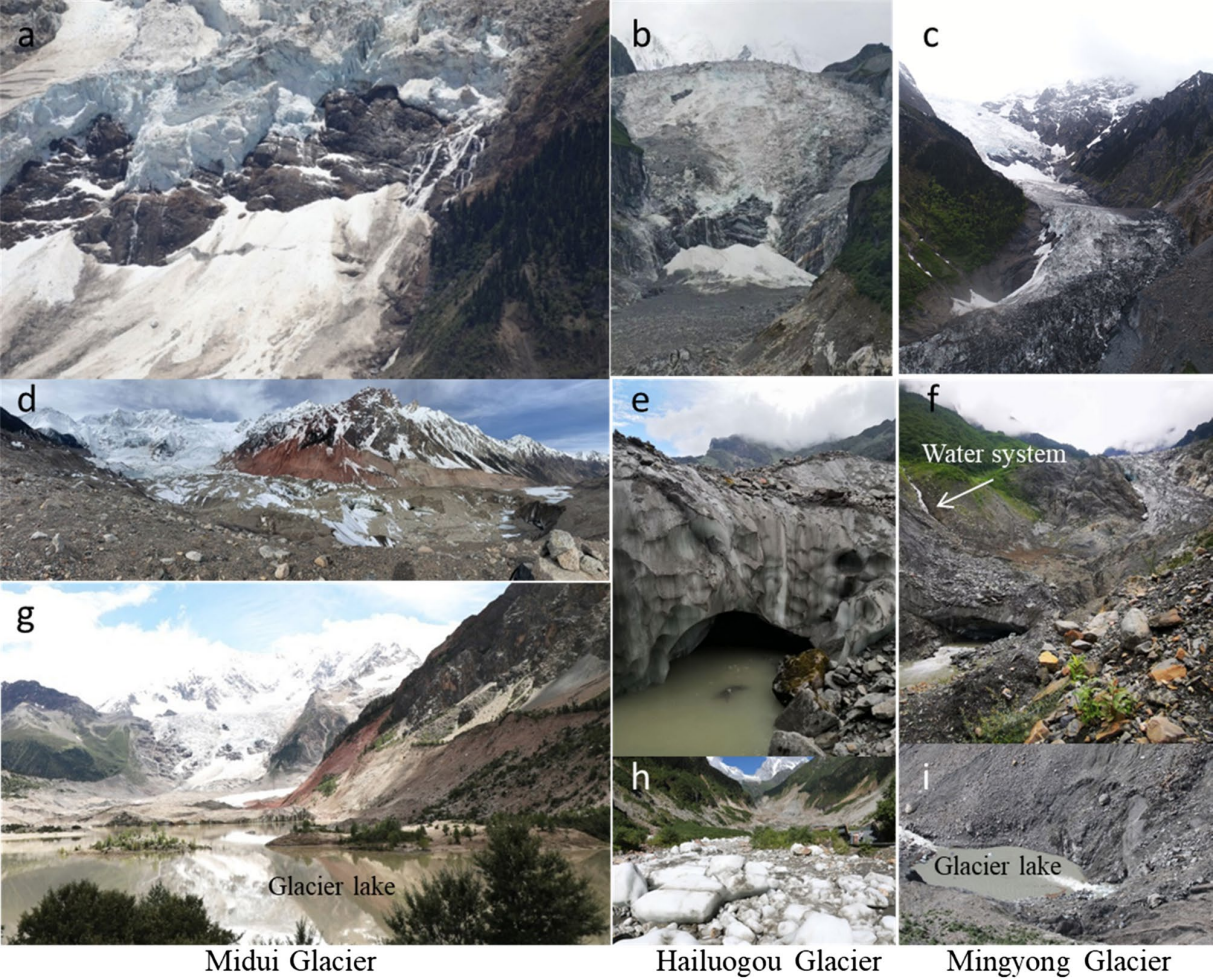
Some new features of rapid changes in temperate glaciers. (**a**,**b**,**c**) the separation of glacier accumulation area and melting area: (**d**) is a collapsed landform of the ice surface caused by the outburst of water systems within the ice body; (**e**,**f**) is two outlets at glacier terminal; (**g**,**i**) is two moraine lakes of glacier terminal; (**h**) shows a large amount of remaining ice blocks formed by the outburst of water system within the ice body).

Solid precipitation in winter and spring is the main way in which glacier mass accumulates, while liquid precipitation in summer and autumn fitted into the monsoon climate, high temperature and surface moraine cover accelerate the melting of glaciers, which are the general characteristics of temperate glaciers^8^. Temperate glaciers have high slopes, the ice surface is covered by the moraine, the ice surface is severely fragmented, and the temperature of ice body is higher and ice flow velocity is faster. In recent years, temperate glaciers have also shown other unique characteristics. Due to the rapid melting of glaciers, accumulation and melting areas of some typical temperate glaciers have been separated (Fig. 6a,b,c), and glacier terminal appeared collapse-like mass loss phenomenon.

At the same time, some glaciers have the phenomenon of water system collapse within the ice body or the water systems on both sides of the valley have entered the ice body (Fig. 6d,f), which accelerates the rapid retreat of such glaciers, and often leads to the formation of outlets and moraine lakes at the end of the glacier (Fig. 6e,g,h,i), increasing the risk of glacial lake outburst floods. Overall, temperate glacier change was more significant to the air temperature than cold glaciers^3,8,56^. These new characteristics in the process of rapid change of temperate glaciers also accelerated the mass loss of glaciers.

### Correlation between temperature, precipitation and mass balance

Because of the geographic differences of the different glacier locations, glacial scales, glacial local climate, and water vapor resources, the glacial changes in these different regions also are different. Existing studies have revealed the relationship between climate change and temperate glacier advancement and retreat in the past 100 years. The results show that cold and dry climate corresponds to the advancing or stable stage of temperate glaciers, and warm and humid climate corresponds to the retreat and loss stage of temperate glaciers, which fully reflects the influence of temperature on temperate glaciers greater than precipitation^15,16^.

**Figure 7:**
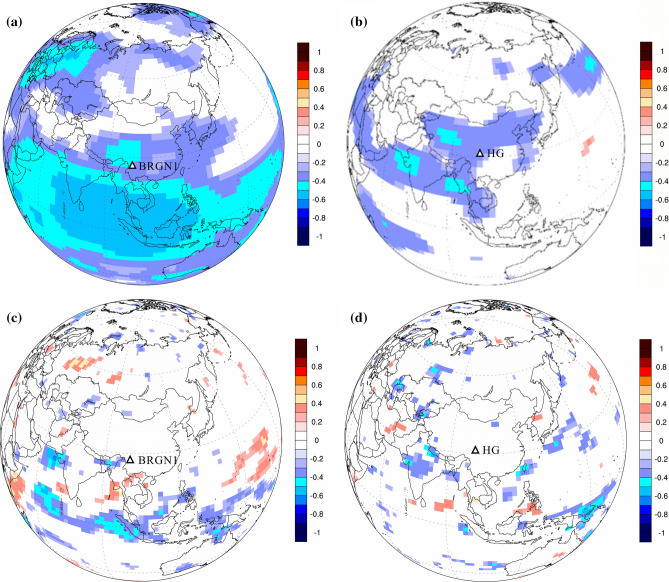
Spatial correlation (≥ 95% confidence level) between 500 hPa temperature and mass balance in Baishui River Glacier No. 1 (BRGN1) (**a**, 1958–2016) and Hailuogou Glacier (HG) (**b**, 1959–2008), and total precipitation and mass balance in the BRGN1) (**c**) and HG (**d**) (the bars in the figure represent correlation). The maps were created by NCL Version: 6.6.2 (http://www.ncl.ucar.edu/citation.shtml/).

To verify the relationship between mass balance of temperate glacier and regional temperature and precipitation fields, the study took BRGN1 and HG as two typical temperate glaciers with longer mass balance data, to reveal their spatial correlation with temperature and precipitation. The result showed that both the mass balance in the BRGN1 and HG are negatively correlated to temperature in southwest China (R = − 0.5, 99% confidence level) (Fig. 7a,b), while there is no correlation between the mass balance and regional precipitation (Fig. 7c,d). The spatial correlation pattern indicates that annual mass balance in temperate glacier is more sensitive to regional temperature than precipitation. Of course, this rule or inference still needs to be re-confirmed in future study.

## Conclusions and prospects

The mass loss of China’s temperate glaciers was much higher than that of continental and polar continental glaciers. Among the glaciers studied, the reduction rates of glaciers in Kangri Garpo, DSM, YSM, and MSM were much higher than the national average. The length of the temperate glacier retreat was equally severe. The recent length retreat rate of the Azha Glacier, Kangri Garpo, Mingyong Glacier, MSM, HG, GSM, BRGN1, and YSM was much higher than that of other regions. Consistent with the glacier length retreat rate, the temperatures also changed at a faster rate^6,8,33,57^. The mass balance of the temperate glaciers in China was the most dramatic. The annual mass balance from the HG, BRGN1, PRGN 4, 10, 12, 94, 390, and Demula Glacier fluctuated between − 2.48 and 0.44 m w.e.

China’s temperate glaciers are controlled significantly by the South Asian monsoon system, the precipitation is abundant, the summer mist is lingering, and the winter is covered with snow. At the same time, temperate glacier regions have high terrain, high mountains, and deep valleys, and most of the glaciers are covered with surface ridges, which form shadows that are difficult to eliminate on remote-sensing images. Temperate glaciers have complex topography and weather conditions, which make traditional observation methods extremely difficult, pose personal safety risks, and affect the wide application of remote-sensing technology. In the future, we will continue to strengthen ground monitoring using unmanned aerial vehicle (UAV) technology and high-resolution radar data (e.g., SAR and InSAR) to automate and digitize glacier monitoring. In addition, small and midsize UAV observation technology will be used in glacier orthophoto acquisition, digital surface model construction, and large-scale topographic map production to monitor ice flow. Synthetic aperture radar technology will be used to determine glacier thickness and ice surface topography. These tools have significant potential to compensate for the shortcomings of ground observations and satellite remote sensing. They will provide technical support to improve observations of the mass balance of traditional glaciers and will address the difficulty in assessing glacier mass balance at a large regional scale. In addition, it is essential to be able to comprehensively analyze the impact of rapid glacial changes on water resources and regional tourism and then propose adaptive management countermeasures.

## Supplementary Information

Supplementary information.

## Abbreviations

QTP: Qinghai-Tibet Plateau
TAR: Tibet Autonomous Region
MSM: Meili Snow Mountain
GSM: Gongga Snow Mountain
YSM: Yulong Snow Mountain
DSM: Dagu Snow Mountain
HG: Hailuogou Glacier
BRGN 1: Baishui River Glacier No. 1
PRGN 4: Parlung River Glacier No. 4
PRGN 10: Parlung River Glacier No.10
PRGN 12: Parlung River Glacier No.12
PRGN 94: Parlung River Glacier No. 94
PRGN 390: Parlung River Glacier No. 390
