# Supplementary material for: Spatiotemporal dynamic characteristics of typical temperate glaciers in China
Source: Sci Rep. 2021 Jan 12;11:657. doi: 10.1038/s41598-020-80418-7 (PMC7804466; doi:10.1038/s41598-020-80418-7)
Supplement: Supplementary file 1 — Supplementary information. [file 41598_2020_80418_MOESM1_ESM.pdf]

Spatiotemporal Dynamic Characteristics of Typical Temperate Glaciers in China

Wang Shijin, Che Yanjun, Wei Yanqiang

Supplementary Table. The information of topographic maps and remote sensing images used in this study

Data Type	Name or code	Date	Scale/Spatial resolution of used
Topographic maps	Longhua 7-47-33-C	1957	1:500000
Topographic maps	9-48-134	1966	1:1000000
Topographic maps	8-47-128、8-47-85-182	1971	1:1000000
TOPO DEM	H47e016002-5/H47e017002-5/H47e018002-5	1980	30m
Landsat MSS	LM21400381975259GDS03	1975.9.16	60m
Landsat5 TM	LT05_L1TP_131038_19891025_20170214_01_T1	1989.10.25	30m
Landsat7 ETM+	LE07_L1TP_131038_20000812_20170210_01_T1	2000.8.12	30m
Landsat7 ETM+	LE07_L1TP_131038_20111014_20161205_01_T1	2011.10.14	30m
Landsat8 OLI	LC08_L1TP_131038_20171006_20171014_01_T1	2017.10.6	30m
Landsat TM	LT51340402001352BJC00	2001	30m
Landsat TM	LT51340402002003BJC00	2002	30m
Landsat OLI	LC81330402015272LGN00	2017	15m
LandSat5	134/40	1987	30m
LandSat5	134/40	2000	30m
LandSat7	134/40	2009	30m
LandSat8	134/40	2017	30m
Google Earth	134/40	2014	-
LandSat5 TM	LT513104119940905BJC00	1994	30 m
LandSat7 ETM+	LE713104120080311SGS00	2008	30 m
Google Image	11-55-03.tif	2015-11-22	1.2 m
LandSat8 OLI	LC813104120170413LGN00	2013	30 m
LandSat8 OLI	LC813104120131011LGN00	2017	30 m
Landsat TM	132/40	1974	30m
Landsat 7 ETM+	132/40	2001	30m
LandSat8 OLI	132/40	2013	30 m
Landsat MSS	131/39	1974	30m
Landsat MSS	131/39	1989	30m
Landsat7 TM	131/39	2009	30m
LandSat8 OLI	131/39	2016	30m
